# Cyclic stretch induced oxidative stress by mitochondrial and NADPH oxidase in retinal pigment epithelial cells

**DOI:** 10.1186/s12886-019-1087-0

**Published:** 2019-03-18

**Authors:** Xida Liang, Zengyi Wang, Meng Gao, Shen Wu, Jingxue Zhang, Qian Liu, Yanping Yu, Jing Wang, Wu Liu

**Affiliations:** 1 Beijing Tongren Eye Center, Beijing Tongren Hospital, Capital Medical University; Beijing Ophthalmology & Visual Sciences Key Laboratory, Beijing, 100730 China; 2 Beijing Institute of Ophthalmology, Beijing Tongren Eye Center, Beijing Tongren Hospital, Capital Medical University; Beijing Ophthalmology & Visual Sciences Key Laboratory, Beijing, 100730 China

**Keywords:** Cyclic stretch, RPE, Oxidative stress, Age-related macular degeneration

## Abstract

**Background:**

Vitreomacular adhesion (VMA) has been reported to associated with age-related macular degeneration (AMD). Understanding the mechanisms underlying cyclic stretch induced in retinal pigment epithelial cells (RPE) may be important for the treatment of VMA-related AMD.

**Method:**

Cyclic stretch (1HZ, 20% elongation) was applied to cultured ARPE-19 cells for 15 min, 2 h, 6 h, 12 h, 24 h by flexcell FX-5000 Tension system. Total reactive oxygen species (ROS) were detected using DCFH-DA. Mitochondrial superoxide were detected using MitoSOX Red mitochondrial superoxide indicator. NADPH oxidases (NOX) and signaling pathways, such as p38 and PKC, were detected using western blot. Apocycin (Apo) were used as NOX inhibitors.

**Result:**

High levels of total ROS were detected from 15 min to 24 h, whereas mitochondrial superoxide were higher only in early time. NOX2 were significantly increased at 24 h. NOX4 were significantly increased at 2 h and reach its peak at 24 h. P-p38 was significantly increased at 12 h and 24 h. P-PKC was significantly increased at 15 min and kept a persistent high level. The upregulated expression of NOX4 by cyclic stretch can be significantly decreased under p-PKC inhibitor other than p-p38 inhibitor.

**Conclusion:**

Cyclic stretch induce oxidative stress from both mitochodrial and NADPH oxidase in RPE cells, which may prompt oxidative damage in VMA-related AMD.

## Background

Age-related macular degeneration (AMD) is a progressive chronic retina disease and a leading cause of vision loss worldwidely [1]. The pathogenesis is complicated and not thoroughly explained. Among these, oxidative damage caused by oxidative stress contribute to both the onset and progression of AMD. Although variety pathogenesis including hereditary susceptibility, inflammation, neovascularization, autophagy have relationship with AMD, oxidative damage appears to be a hallmark of early AMD and combine with other pathogenesis to progress to the pathology and visual morbidity associated with advanced AMD [2]. Recent clinical studies have found that vitreomacular adhesion (VMA) may be risk factors associated with the AMD [3]. A systematic review reported in 2013 showed that, the prevalence of vitreomacular adhesion in wet-AMD was 2.15 times that of controls [4]. VMA also significantly influence the treatment of anti-VEGF for wet-AMD patients, which lead to a poorer vision prognosis, slower retinal thickness recovery and more injection times [5–8]. Ocriplasmin, a drug to relieve the VMA, can reduce the injection times of anti-VEGF drug in wet-AMD patients [9]. The VMA, which means an anomalous attachment between vitreous cortex and retinal surface, may impact the chorioretinal interface [10]. Robison et al. suggests that the VMA may cause chronic and continuous traction on the macula and may promote the progression of wet-AMD [10]. Besides, the formation of pigmental epithelium detachment (PED) also provide stress to RPE cells. Therefore, we hypothesis that cyclic stretch may impair the physiological state of retinal cells and participate in the pathogenesis of AMD.

Retinal pigment epithelium (RPE), which lies beneath the photoreceptor cells, plays critical roles in the pathogenesis of AMD [11]. In our previous studies, cyclic stretch can induce changes in morphological, actin cytoskeleton and up-regulate cytokines in RPE cells, which finally cause apoptosis [12, 13]. However, the early changes and specific mechanism remains unclear. Previous studies have indicated that mechanical stress can induce and exacerbate the oxidative damage. Wedgwood et al. found that cyclic stretch increases the level of ROS from mitochondrial and NOX4 signaling in pulmonary artery smooth muscle cells [14]. Li et al. found that excessive cyclic tensile strain over 12% elongation could break the balance of oxygen free radical system and lead to cytotoxicity [15]. Wang et al. found that moderate stretch can decrease the plasma membrane integrity and mitochondrial activity in SH-SY5Y cells [16]. Davidovich et al. found that cyclic stretch can induce oxidative stress and alter the barrier properties via NF-kB pathway and ERK activation in alveolar epithelium [17]. As far as we know, no research has focused on whether cyclic stretch can induce the oxidative stress on RPE cells.

The purpose of this study is to determine if cyclic stretch could induce the ROS generation in RPE cells. After identify the ROS generation by cyclic stretch, we explore the origin are both mitochondrial and NADPH oxidase. Then, we found that the ROS generated by mitochondrial and NADPH oxidase has different peak time. At last, we explore the expression level of PKC phosphorylation and p38 phosphorylation. In conclusion, cyclic stretch induce the oxidative stress through both mitochondrial and NADPH oxidase in RPE cells, which has different activation time and combined to form a persistence oxidative damage.

## Methods

### Culture of ARPE-19

ARPE-19 cells were from the ATCC cop (pruchased by Dr. Shen Wu). The culture medium consisted of DMEM/F12 medium (Gibco) supplemented with 10% FBS (Gibco), 100 U/ml penicillin (Gibco) and 100μg/ml streptomycin (Gibco) at 37 °C in a humidified atmosphere of 95% air and 5% CO_2_. Cultured medium was replaced and cells were passaged as necessary.

### Cyclic stretch

Cells were passaged to DMEM containing 10% FBS six-well BioFlex plates coated with collagen type I (Flexcell) for 24 h to form a confluent monolayer. Then the medium was replaced by DMEM without FBS for 6 h before stretch. A Flexcell FX-5000TM Tension System (Flexcell International Corporation, Burlington, CA, USA) was used to order cyclic mechanical stretch. Cells were stretched at a frequency of 1HZ with 20% amplitude and a 1:1 stretch:relaxation ratio for 15 min, 2 h, 6 h, 12 h and 24 h. Both the tension system and the control plates were placed in the incubator at 37 °C, with 5% CO_2_.

### Detection of total ROS and mitochondrial superoxide

A broad spectrum of ROS were detected in living cells using the DCFH-DA (Beyotime). Dilute the 10 mM DCFH-DA in medium without FBS to make a 10 μM DCFH-DA working solution. Apply 2 mL of 10uM DCFH-DA to cover cells for 20mins at 37 °C, protected from light. Wash cells gently three times with phophate buffered saline (PBS). Mitochondrial superoxide was detected in living cells using the fluorogenic dye MitoSOX (invitrogen). Dissovle the 50μg MitoSOX component in 13uL of Dimethyl sulfoxide (DMSO) to make a 5 mM MitoSOX reagent stock solution. Dilute the 5 mM mitoSOX reagent stock solution in medium without FBS to make a 5 μM mitoSOX reagent working solution. Apply 2 mL of 5uM mitoSOX reagent working solution to cover cells for 10mins at 37 °C, protected from light. Wash cells gently three times with PBS. Then the cells were covered with 1μg/ml Hoechst staining solution (Thermofisher). The cells were observed by using a confocal laser scanning microscope (Leica TCS-NT, Germany). The acquired images were converted into binary images for the quantification of the average fluorescence intensity using ImageJ software (National Institutes of Health, USA).

### Western blot analysis

Proteins were extracted by RIPA lysis buffer (Cell Signaling Technology, USA) supplemented with protease inhibitor cocktail (CWBIO, China) and Phosphatase Inhibitor Cocktail (CWBIO, China). Total concentration were measured by a BCA protein assay kit (CWBIO, China). 8, 10, 12% polyacrylamide gels by Sodium dodecyl sulphate-polyacrylamide gel electroporesis (SDS-PAGE) were made according to the molecular weight of the proteins. Then the gels were blotted onto polyvinylidene difluoride (PVDF) membranes and blocked with 5% non-fat milk. The membrane were incubated with primary antibodies: NOX2 (1:1000, abcam, No. ab80508), NOX4 (1:1000, abcam, No. Ab133303), p38 MAPK (1:1000, CST, No. 8690), p-p38 MAPK (1:1000, CST, No. 4511), p-p38 blocking peptide (1:200, CST, No. 1170), p-PKC (1:1500, CST, No. 2060), Staurosporine (p-PKC inhibitor, CST, No. 9953), GADPH (1:1000, CST, No. 5174). GSH (Beyotime, No. S0073) is an antioxidant agents that can eliminate free radials. Apocynin (APO, Selleck, No. S2425) is discovered as an inhibitor of NOXs. The primary antibodies were diluted in TBST with 5% non-fat milk and incubated with the membranes overnight at 4 °C. Then, the membranes were washed 3 times with Tris-Buffered Saline and Tween 20 (TBST) and incubated with a secondary goat anti-rabbit lgG antibody conjugated to horseradish peroxidase (1:1000, CST, No. 7074) for 1.5 h at room temperature. Finally, the chemiluminescence signals were visualized using an ECL imager (Millipore, USA) and analysed using BIO-RAD Quantity One Imaging software (Bio-Rad, USA). Western blot was normalized to GADPH. Data are shown as fold relative to control lambs.

### Statistical analysis

All data were expressed as the mean ± SEM. At least three samples of each condition were examined. One-way ANOVA and Bonferroni’s multiple comparison test were used to determine statistical significance (SPSS 22.0). Statistical significance of differences between two groups was determined using a two-tailed paired t-test. Data were deemed statistically significant at *P* ≤ 0.05.

## Result

### Cyclic stretch increases the expression levels of total ROS in ARPE-19

**Fig. 1:**
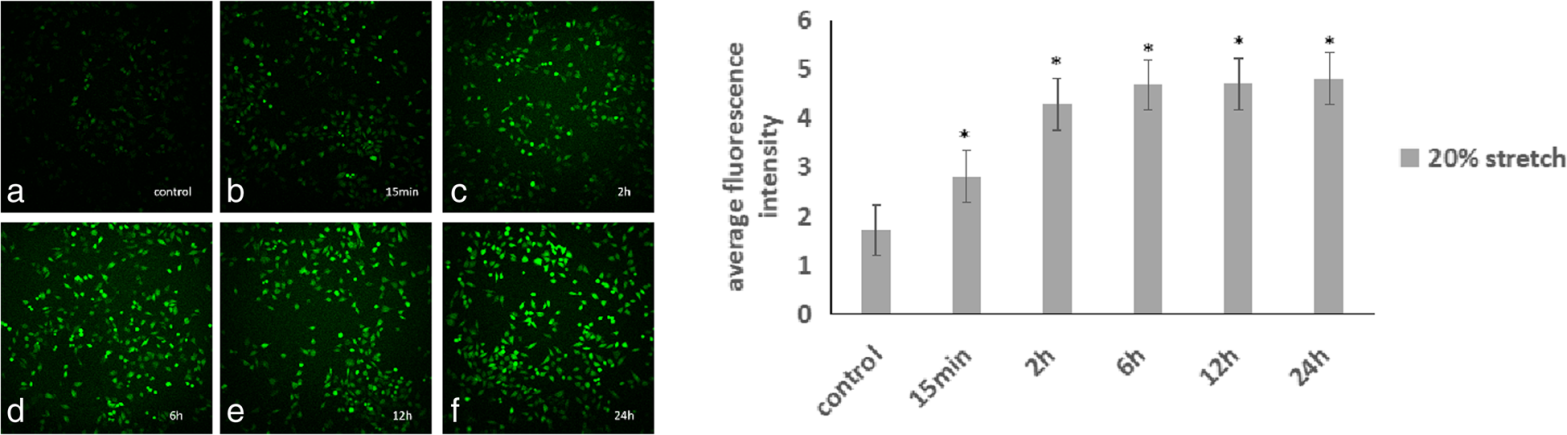
Total ROS expression stained with DCFH-DA under cyclic stretch in ARPE-19 cells. a-d The total ROS expression was significantly increased at 15 min by cyclic stretch (*p* < 0.01). The total ROS expression at 6 h was significantly increased compared to both the control(*p* < 0.01) and the 15 min group(*p* < 0.01). There was no significant difference between the 6 h, 12 h and 24 h group(*p* = 0.952, 0.835,0.800). Bar = 10 μm

Under 20% elongation cyclic stretch, the expression levels of total ROS were significantly increased at 15 min (*p* < 0.01). The total ROS expression at 6 h was significantly increased compared to the 15 min group(p < 0.01). There was no significant difference between the 6 h, 12 h and 24 h group(*p* = 0.952, 0.835,0.800). The expression of total ROS started to increase at 15 min and reach its peak at 6 h. (Fig. 1).

### Cyclic stretch increases the expression levels of NADPH oxidase in ARPE-19

**Fig. 2:**
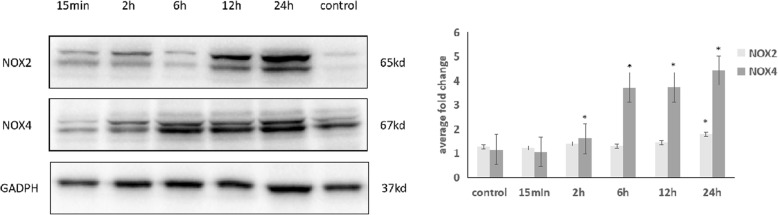
NOX2 and NOX4 expression under cyclic stretch in ARPE-19 cells based on Western blotting. The NOX2 expression was significantly increased at 24 h by cyclic stretch (*p* = 0.020). There was no significant difference between the control, 15 min, 2 h, 6 h, 12 h group(*p* = 0.851, 0.586, 0.811, 0.453). The NOX4 expression was significantly increased at 2 h, 6 h, 12 h and 24 h by cyclic stretch (*p* < 0.01,< 0.01,< 0.01,< 0.01). There was no significant difference between the control and the 15 min group(*p* = 0.316)

Under 20% elongation cyclic stretch, the expression levels of NOX2 were significantly increased at 24 h (*p* = 0.20). There was no significant difference between the control, 15 min, 2 h, 6 h and 12 h group(*p* = 0.851, 0.586, 0.811, 0.453). The NOX4 expression was significantly increased at 2 h, 6 h, 12 h and 24 h by cyclic stretch (*p* < 0.01,< 0.01,< 0.01,< 0.01). There was no significant difference between the control and the 15 min group(*p* = 0.316). (Fig. 2).

### The expression levels of mitochondrial superoxide anion increases after early cyclic stretch in ARPE-19

**Fig. 3:**
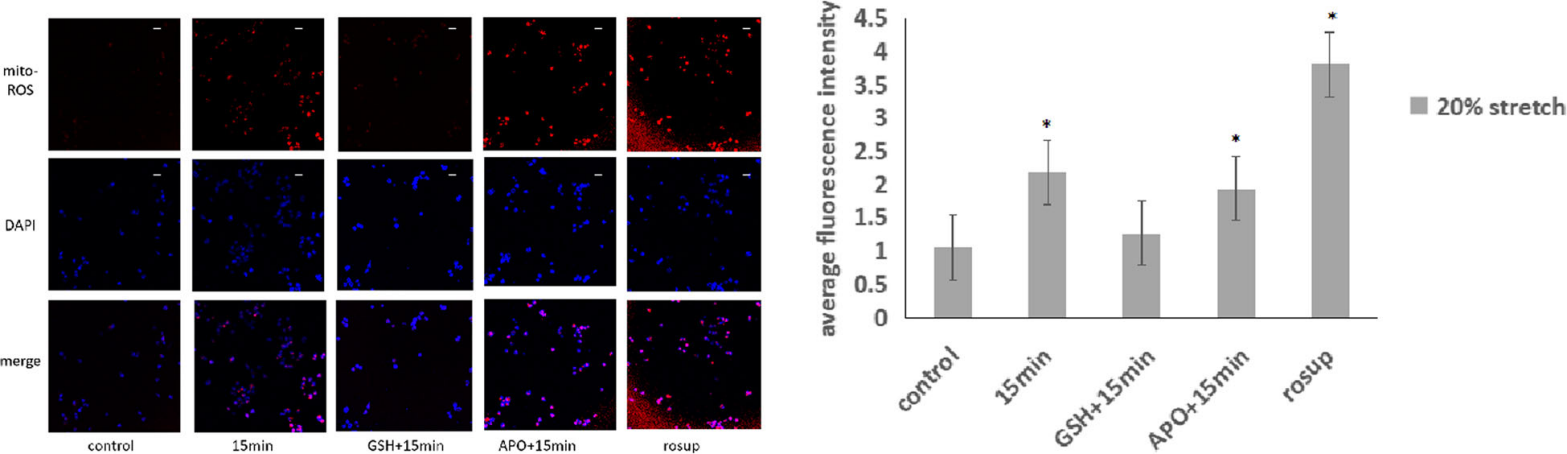
Mitochondrial superoxide anion expression stained with Mito-SOX under cyclic stretch in ARPE-19 cells. The mitochondrial superoxide expression was significantly increased at 15 min by cyclic stretch (*p* < 0.01). The GSH + 15 min group has no significant difference with the control group(*p* = 0.278). The APO + 15 min group has significant difference with the control group(*p* < 0.01).Bar = 10 μm

In order to find whether the mitochondrial system participated in the oxidative stress caused by cyclic mechanical stretch, we detect the mitochondrial superoxide level in RPE cells. Under 20% elongation cyclic stretch, the expression levels of mito-SOX were significantly increased at 15 min. Then, we form the control group, 15 min cyclic stretch group, GSH + 15 min stretch group, APO + 15 min stretch group and the rosup group. The mitochondrial superoxide expression was significantly increased at 15 min by cyclic stretch (*p* < 0.01). The GSH + 15 min group has no significant difference with the control group(*p* = 0.278). The APO + 15 min group has significant difference with the control group(*p* < 0.01). The result showed that the up-regulated mitochondrial superoxide level can reduced by GSH, but can’t reduced by APO. (Fig. 3).

**Fig. 4:**
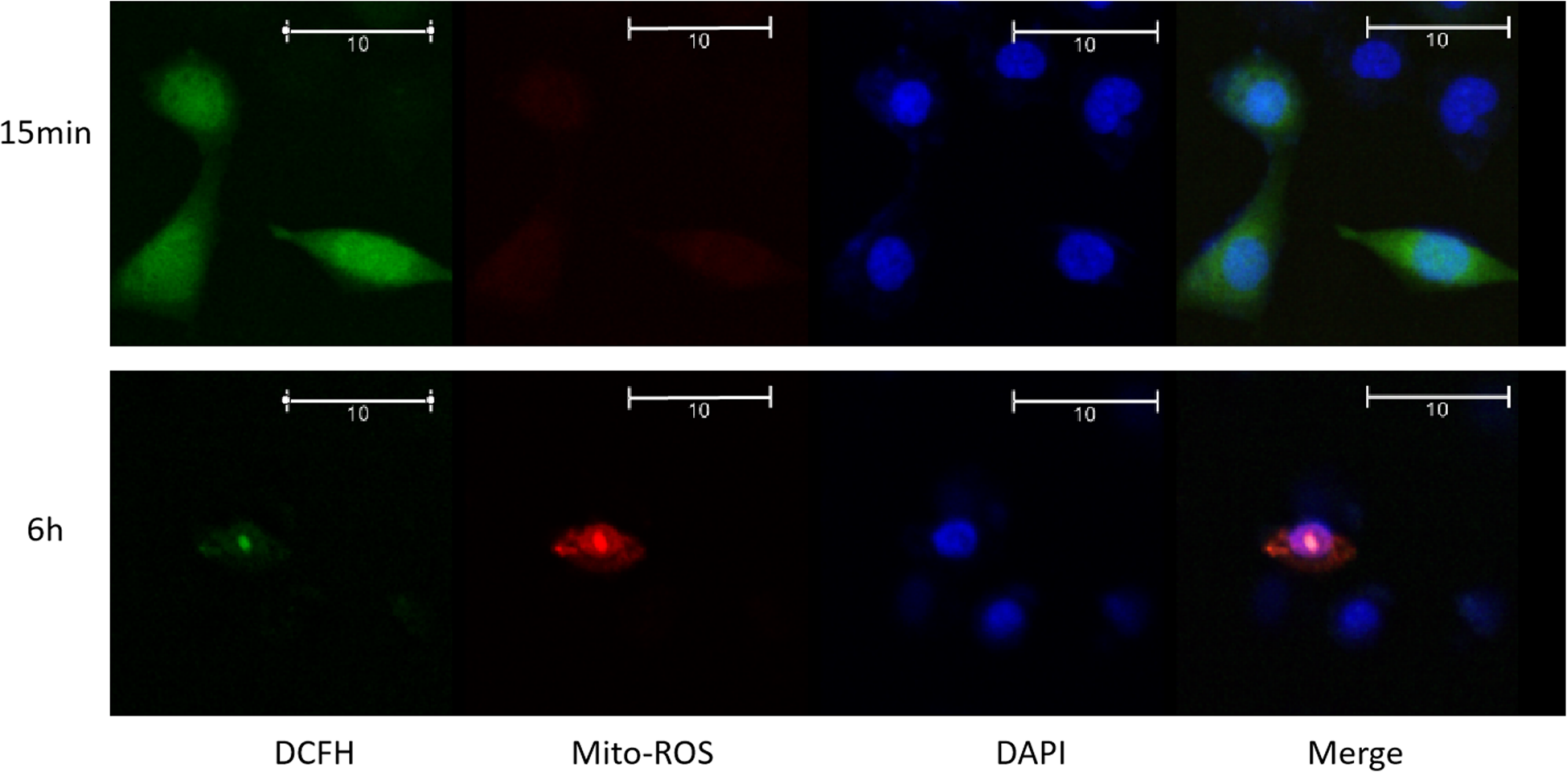
Double stained with DCFH-DA and Mito-SOX under cyclic stretch in ARPE-19 cells. The mitochondrial superoxide expression at 6 h was significantly decreased compare to at 15 min (*p* < 0.01) by cyclic stretch . The total ROS expression at 6 h was significantly increased compare to at 15 min(*p* < 0.01) by cyclic stretch

The previous result suggests that the NADPH oxidase and mitochondrial system have different starting time. With the DCFH-DA/Mito-SOX double staining double, we observed the RPE cells at 15 min and 6 h after cyclic stretch. We found that the mito-SOX increased significantly at 15 min and relatively decreased at 6 h (*p* < 0.01), whereas the total ROS increased continuously.(Fig. 4).

### Cyclic stretch increase the expression levels of p-p38, p38, p-PKC

**Fig. 5:**
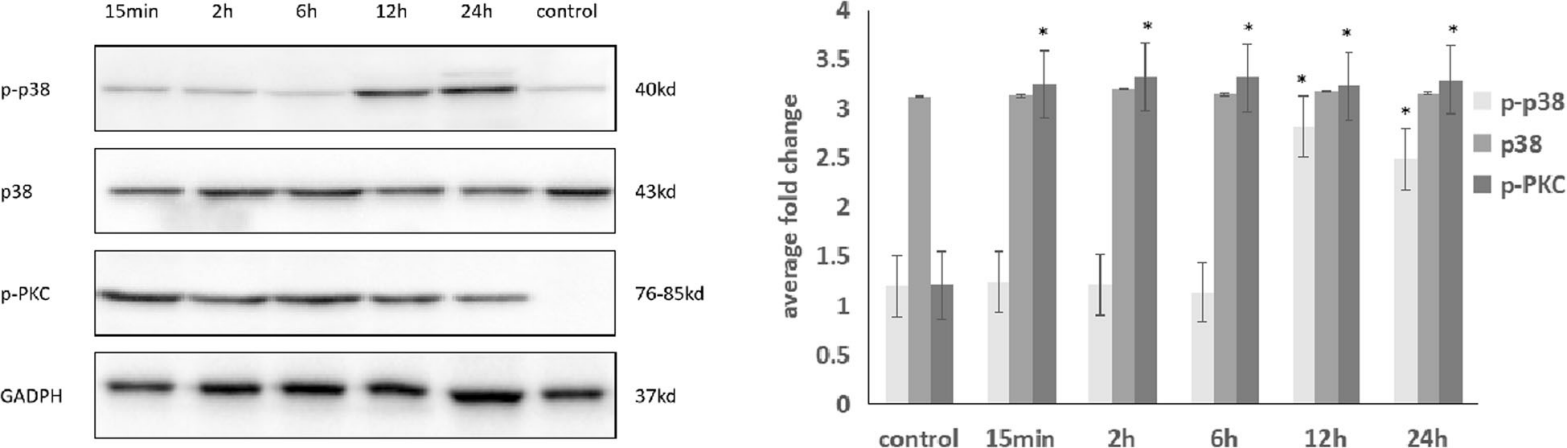
P-p38, p38 and p-PKC expression under cyclic stretch in ARPE-19 cells based on Western blotting. The p-p38 expression was significantly increased at 12 h and 24 h by cyclic stretch (*p* < 0.01,< 0.01). The p-PKC expression was significantly increased at 15 min, 2 h, 6 h, 12 h and 24 h by cyclic stretch (*p* < 0.01, < 0.01, < 0.01, < 0.01, < 0.01)

**Fig. 6:**
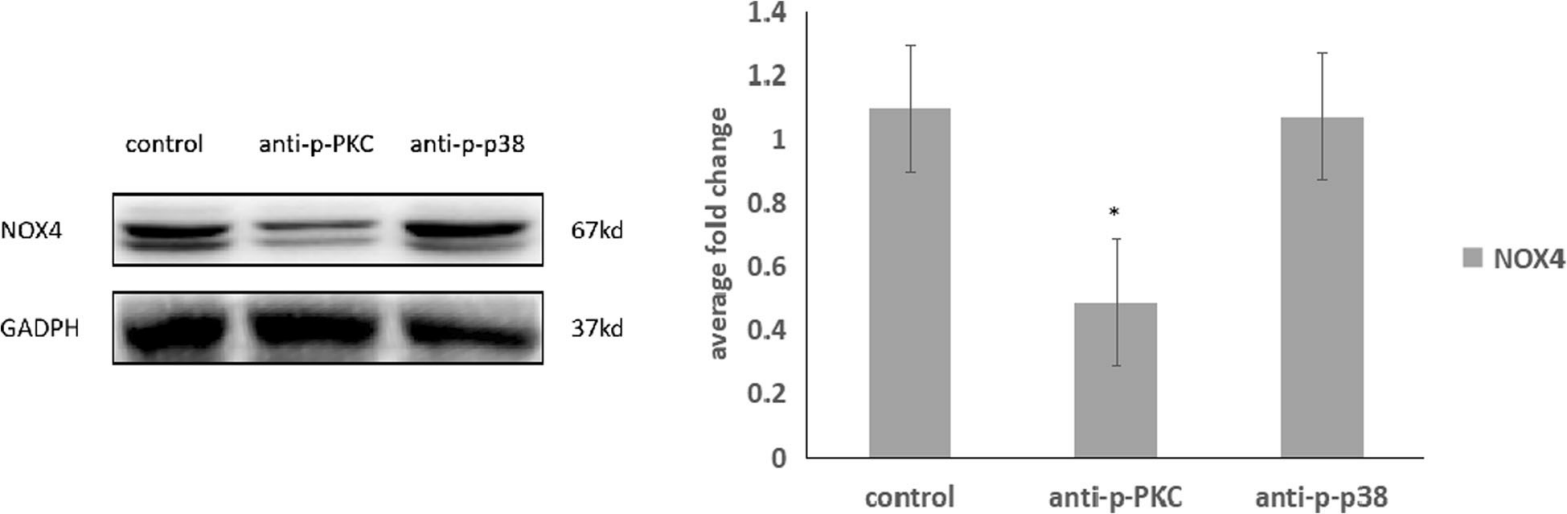
NOX4 expression treated with p-PKC and p-p38 inhibitor under cyclic stretch in ARPE-19 cells based on Western blotting. The NOX4 expression was significantly decreased by inhibitor of p-PKC(*p* = 0.036), and not significantly influenced by inhibitor of p-p38 (*p* = 0.844)

Under 20% elongation cyclic stretch, the protein expression levels of phosphorylate p38 were significantly increased after 12 h (p < 0.01). Under 20% elongation cyclic stretch, the protein expression levels of pPKC were significantly increased after 15 min (p < 0.01). We found that the p38 and pPKC were both activated by cyclic stretch, and the pPKC seems to be activated in the early time (Fig. 5). Therefore, we detacted the NOX4 expression level under treatment of inhibitor of p-p38 and p-PKC and cyclic stretch for 24 h. We found that the NOX4 expression was decreased by inhibitor of p-PKC(*p* = 0.036), and not significantly influenced by inhibitor of p-p38(*p* = 0.844) (Fig. 6).

## Discussion

Oxidative stress, which is recogonized as the initiation factor in AMD. It’s believed that oxidative changes are a hallmark of early AMD [2], and dietary anti-oxidant supplementation slows progression of the disease [1]. The oxidative damage of AMD is usually identified as senescence proformance, but whether there exists other triggers remains unclear. Recent clinical researches found that VMA is related with AMD, which may caused by chronic stretch formed by VMA [10]. In this study, we found that mechanical stretch can induce oxidative stress of RPE cells in less than 15 min and keep rising to a high level, by both mitochondrial and NOXs pathway.

In our study, 20% elongation cyclic stretch induce the expression of total ROS in less than 15 min, and keeps a persistently high level at 6 h. Previous studies have focused on the relationship between cyclic stretch and blood vessel diseases, since the blood vessels are constantly exposed to mechanical stresses. Birukov et al. believed that ROS signaling induced by cyclic stretch plays an essential role in physiologic regulation of vascular function and vasucular remodeling [18]. In pulmonary arterial smooth muscle cell, Greenwood et al. found that cyclic stretch can increase VEGF expression due to the up-related ROS induced by NADPH oxidase [19]. In Seko Y’s study, mechanical stress can induce the expression of VEGF in RPE of rat in vitro [20]. The effect of cyclic stretch on the expression of ROS and the pathways induced in RPE cells has not been studied before. However, the up-regulated of ROS by cyclic stretch has already been found in many other cell types, such as pulmonary artery smooth muscle cell, bone marrow mesenchymal stem cell, neonatal cardiomyocyte, type II alveolar epithelial cell et al. [14–17]. In their studies, 20% elongation and 1 HZ cyclic stretch were widely used in the mechanical stretch model using Flexcell, since Chiu et al. observed the greatest effect on protein expression at 20% stretch compared with 10% stretch [21]. However, whether the paremeter can exactly mimic the situation in human eye need to be further studied. In Davidovich’s study, cyclic stretch up-regulated the general ROS, which increases the pulmonary alveolar epithelial permeability [17]. It seems that oxidative stress caused by cyclic stretch can be an initiator, which can activate celluar signaling, regulate gene expression and change the physiological and pathological activities.

In our study, the NOX2 and NOX4 expression was significantly increased by cyclic stretch. In general, the origin of ROS generation contains mitochondria, NADPH oxidase, peroxisomes, cytochrome P-450, et al. Among these, NAPDH oxidase is the only system which generate ROS as the primary and maybe the unique function. The NOX familay have seven main members, including NOX1, NOX2, NOX3, NOX4, NOX5, DUOX1 and DUOX2. Among these, NOX2 and NOX4 are two spectral members and have been extensively studied. NOX2 has been demonstrated can be activated by interferon-γand angiotensin II [22],and can be induced through a CHOP/CAMKII pathway to mediate apoptosis [23].NOX4 was initially identified expressed in the kidney, and then was found to be constitutively activated in various organs and cells. NOX4 is induced early during the UPR and is upstream to several signaling events that may induce autophagy, survival, or eventually apoptosis [23]. This indicates that the up-regualted NOX2 and NOX4 may participate in the apoptosis after long-term cyclic stretch in our previous study [12].

Mitochondria represents a major source of endogenous reactive oxygen species (ROS) in RPE [24, 25]. In physiological state, RPE cells survived from high oxidative stress caused by phagocytosis of photoreceptor outer segments and blue light injury, which due to its elevating cellular antioxidants and a higher nuclear DNA (nDNA) repair capacity [26]. However, the increased ROS may anchor on mitochondria and exhibit damage to mitochondrial DNA (mtDNA), which appears to be relatively hard to repair than nuclear DNA. This make the mitochondria to be the “weak link” in RPE cells protecting from oxidative stress [25]. The damaged mtDNA increased the ROS level which form a “vicious circle” and induce the oxidative damage. In our result, mitochondria superoxide was significantly upregulated by cyclic stretch. This phenomenon can be totally diminished by GSH, but only partly neutralized by NOX inhibitors. This suggests that the two origin of ROS are relatively independent, which can’t entirely replaced by each other.

In our result, the superoxide produced by mitochondrial upregulated in less than 15 min and strated to down regulated in 6 h. This may due to the instability of superoxide. Superoxide is known to be the initial ROS produced by mitochondrial respiratory chain [27], which can be easily converted into H2O2 by dismutation of manganese superoxide dismutase (MnSOD) and OH^.^ by fenton chemistry. H2O2 and OH^.^ are relatively stable ROS and responsible for many sites of oxidative damage, including mtDNA and nDNA. A noteworthy feature is that although superoxide produced by mitochondrial in 6 h is not as much as in 15 min, the total ROS keep a persistent high expression level. This phenomenon indicates that mitochondrial superoxide has converted into other type of ROS in 6 h. In addition, the main products formed by NOX4 is H2O2 other than superoxide [28]. The protein expression level of NOX4 significantly increased after 2 h, and reach its peak at 6 h, which may be the main origin of total ROS at 6 h. Clement et al. proposed that superoxide anion is a mild type of ROS, which could even protect cell death mediated by FAS [29]. Since the continuous transformation from superoxide anions to other types of ROS, this may participate in the apoptosis of RPE cells induced by cyclic stretch, which needs to be identified in the further study.

Cyclic stretch is capable of stimulating release of ROS and activating redox-sensitive signaling pathways [18]. The potential sources including NADPH oxidase system, mitochondrial system, and the xanthine oxidase system. The increased ROS production contribute to the activation of pro-inflammatory transcription factors, activation of growth promoting MAP kinases, upregulation of pro-fibrogenic mediators. In vascular smooth muscle cells, cyclic stretch induced ROS production, led to activation of MAPK signaling including p38, JNK, Erk1/2 and modulated the vascular smooth muscle cell alignment [30]. In cardiac myocytes, high-amplitude cyclic strain induced ROS production, led to activation of JNK and Erk1/2 and induced the appearance of apoptotic phenotype [17]. These studies indicated the role of p38 activation after ROS release by cyclic stretch. In our result, phosphorylate p38 were significantly increased after cyclic stretch for 24 h, which suggests that p38 is involved in mechanical stretch signal. In addition, PKC phosphorylation plays primary role in cellular stress and NOX activation [31]. Also, PKC phosphorylation can activated by several upstream stimuli, which can regulate the NOX activation. In our result, the expression of NOX4 can be decreased by inhibitor of p-PKC, nor the p-p38. This suggests that cyclic stretch may induce the expression of NOX4 through PKC pathway.

Our study still has some limitations. Firstly, whether ARPE-19 cells have RPE cells in vivo in healthy condition is controversial. Besides, whether the exprimental model can mimic the vivo situation needs to be further studied. Care should be taken to interpret these results. Secondly, These experiments were done only by pharmacological inhibitor, not by gene upregulation/down regulation with prasmid/siRNA. Also, the specific mechanism between mitochondrial, NOX and signal pathways needs to be further studied. Thirdly, the mitochondrial superoxide decreases but the total ROS keep rising after cyclic stretch for 6 h. The main element of ROS at 6 h, whether it is H2O2 or other free radials, remains unclear.

## Conclusions

In conclusion, the NADPH oxidases system and mitochondrial system were both activated by cyclic stretch, which induce the release of ROS and cause the oxidative damage in RPE cells. According to our results, the VMA and other mechanical factors may cause oxidative damage, which may promote the course of AMD. Therefore, this research is helpful to explore the pathogenesis of AMD and may provide further suggestions for clinical treatment.

## Abbreviations

AMD: age-related macular degeneration
ANOVA: one-way analysis of variance
Apo: apocycin
ARPE-19: A spontaneously arising RPE cell line derived in 1986 by Amy Aotaki-Keen from the normal eyes of a 19-year-old male who died from head trauma in a motor vehicle accident
BCA: bicinchoninic acid protein assay kit
DCFH-DA: dichlorodihydrofluorescein diacetate
DMEM/F12: Dulbecco’s Modified Eagle Medium/Ham’s Nutrient Mixture F12
FBS: foetal bovine serum
GADPH: Glyceraldehyde-3-phosphate dehydrogenase
MitoSOX: red mitochondrial superoxide Indicator, for live-cell imaging
NOX: NADPH oxidases
PVDF: polyvinylidene difluoride
RIPA: radio immunoprecipitation assay lysis buffer
ROS: reactive oxygen species
RPE: Retinal pigment epithelium
SDS-PAGE: sulfate-polyacrylamide gels
TBST: Tris-buffered saline with 0.05% Tween 20
VMA: vitreomacular adhesion
